# Utilization Trends of SGLT2 Inhibitors in Croatian Clinical Practice: Observational Analysis

**DOI:** 10.3390/medicina62020286

**Published:** 2026-01-31

**Authors:** Andrej Belančić, Marta Kučan Štiglić, Ana Jelaković, Ivan Pećin, Bojan Jelaković, Dinko Vitezić

**Affiliations:** 1 Department of Basic and Clinical Pharmacology and Toxicology, Faculty of Medicine, University of Rijeka, 51000 Rijeka, Croatia; kucanmarta@gmail.com (M.K.Š.); dinko.vitezic@medri.uniri.hr (D.V.); 2 Faculty of Medicine, University of Rijeka, Braće Branchetta 20, 51000 Rijeka, Croatia; anajelakovic9@gmail.com; 3 Department of Nephrology, Arterial Hypertension, Dialysis and Transplantation, University Hospital Center Zagreb, 10000 Zagreb, Croatia; jelakovicbojan@gmail.com; 4 Department of Internal Medicine, Division of Metabolic Diseases, University Hospital Center Zagreb, 10000 Zagreb, Croatia; ivanpecin@yahoo.com; 5 School of Medicine, University of Zagreb, 10000 Zagreb, Croatia

**Keywords:** antihyperglycemic agents, SGLT2 inhibitors, diabetes, heart failure, chronic kidney disease, pharmacoepidemiology

## Abstract

*Background and Objectives:* Sodium-glucose co-transporter 2 (SGLT2) inhibitors have emerged as key agents in the management of type 2 diabetes mellitus (T2DM), with expanding indications in heart failure and chronic kidney disease. This study assessed national trends in SGLT2 inhibitor utilization in Croatia between 2014 and 2024 using data from IQVIA. *Materials and Methods:* Drug use was quantified in defined daily doses per 1000 inhabitants per day (DDD/1000/day), alongside financial expenditure and prescribing patterns. *Results:* Since their market introduction in 2014, SGLT2 inhibitor utilization increased from 0.49 to 11.63 DDD/1000/day by 2024. Fixed-dose combinations with metformin accounted for a growing share of prescribing, reflecting a shift toward adherence-friendly regimens. Dapagliflozin was the most prescribed agent, likely due to broad therapeutic versatility and favorable pricing. Despite these trends, SGLT2 inhibitors (monotherapy) seem to be underutilized, accounting for just 12% of non-insulin antidiabetic prescriptions in 2024. *Conclusions:* These findings highlight the gradual integration of SGLT2 inhibitors into national clinical practice and emphasize the need for targeted educational and policy efforts to overcome therapeutic inertia and align prescribing with evidence-based cardio-renal-metabolic care.

## 1. Introduction

Sodium-glucose co-transporter 2 inhibitors (SGLT2i) represent a pivotal advancement in the management of type 2 diabetes mellitus (T2DM) and, more broadly, in cardio-renal-metabolic care. Four agents (dapagliflozin, empagliflozin, canagliflozin, and ertugliflozin) are currently approved in the European Union, available as monocomponent therapies or in fixed-dose combinations with metformin or dipeptidyl peptidase-4 (DPP-4) inhibitors [1,2,3,4,5]. By selectively inhibiting SGLT2 in the proximal renal tubule, these agents reduce glucose and sodium reabsorption, resulting in glycosuria, natriuresis, and osmotic diuresis. These mechanisms contribute not only to improved glycaemic control but also to favorable haemodynamic effects, such as reduced intraglomerular pressure, preload, and afterload, ultimately translating into benefits for cardiac remodeling and renal function [5].

Clinical trial data and meta-analyses have consistently shown that SGLT2 inhibitors reduce glycated haemoglobin (HbA1c) by approximately 0.5–1% without increasing the risk of hypoglycaemia, regardless of disease duration or β-cell function [6]. The safety profile of this drug class is generally favorable, with predictable adverse effects such as urinary tract infections, vulvovaginal candidiasis, and polyuria being mechanistically linked to their glycosuric action. However, euglycemic ketoacidosis is a rare but serious potential side effect that clinicians should also be aware of [1,2,3,4,5].

Beyond glycaemic control, SGLT2 inhibitors have emerged as cornerstone therapies for heart failure (HF) and chronic kidney disease (CKD), even in patients without diabetes. Landmark cardiovascular and renal outcome trials, including EMPA-REG OUTCOME, DAPA-HF, EMPEROR-Reduced, EMPEROR-Preserved, DELIVER, DAPA-CKD, and EMPA-KIDNEY, have demonstrated substantial benefits across a wide spectrum of clinical endpoints [7,8,9,10,11,12,13]. These findings have redefined the therapeutic scope of SGLT2i and are reflected in contemporary international guidelines [14,15,16,17,18].

Despite the strength of this evidence, underutilization remains a significant concern, particularly among patients with HF or CKD without diabetes. This underuse may reflect persistent therapeutic inertia, limited cross-disciplinary implementation—with prescribing still predominantly concentrated among diabetologists, cardiologists, and nephrologists—economic barriers, underdiagnosis of CKD and HFpEF in primary care, and gaps in familiarity with emerging evidence and updated clinical guidelines [5].

To address these knowledge gaps, the present study evaluates national trends in SGLT2 inhibitor utilization in Croatia from 2010 to 2024, focusing on changes in prescribing patterns and financial expenditures. The findings aim to provide insights into national prescribing practices and inform future healthcare policies regarding T2DM, HF, and CKD management.

## 2. Materials and Methods

This study evaluated total utilization data for all drugs used in diabetes (ATC code A10) in Croatia from 2010 to 2024 and analyzed national utilization trends of sodium-glucose cotransporter-2 (SGLT2) inhibitors from 2014, the year they gained market access, through 2024. Data on pharmaceutical utilization were obtained from IQVIA database, widely recognized source for drug market surveillance and pharmacoepidemiological research. Drug utilization was quantified using defined daily doses per 1000 inhabitants per day (DDD/1000/day), as recommended by the World Health Organization (WHO) Collaborating Centre for Drug Statistics Methodology. The Anatomical Therapeutic Chemical (ATC)/DDD classification system was applied to ensure standardized assessment and facilitate cross-national comparability.

Where WHO-assigned DDD values for specific SGLT2 inhibitors or formulations were revised during the study period, the most recent (2024) DDD assignments were applied retrospectively across the full investigated timeframe to maintain methodological consistency. Fixed-dose combination products were classified in accordance with the 2024 WHO Guidelines for ATC classification and DDD assignment wherein each fixed combination is counted as one DDD, regardless of the number of active pharmaceutical ingredients [19].

Population denominators used in the calculation of DDD/1000/day were obtained from the Croatian Bureau of Statistics. Official census counts were applied for the years 2011 and 2021, while inter-census population estimates published by the Bureau were used for all other years [20].

Expenditure data were reported in euros (€). Total pharmaceutical spending on SGLT2 inhibitors was recorded as absolute annual expenditure, and unit costs were expressed as euros per DDD (€/DDD) (unadjusted for inflation) for each agent.

Temporal trends in drug utilization and expenditure were evaluated using the index of change. This metric was defined as the relative difference between the baseline year and the most recent year (2024), expressed either in DDDs/1000/day or total expenditure (€), depending on the outcome of interest.

## 3. Results

**Figure 1:**
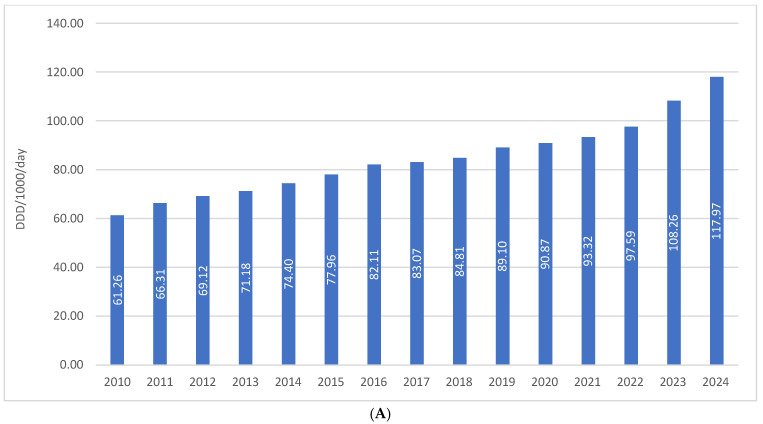

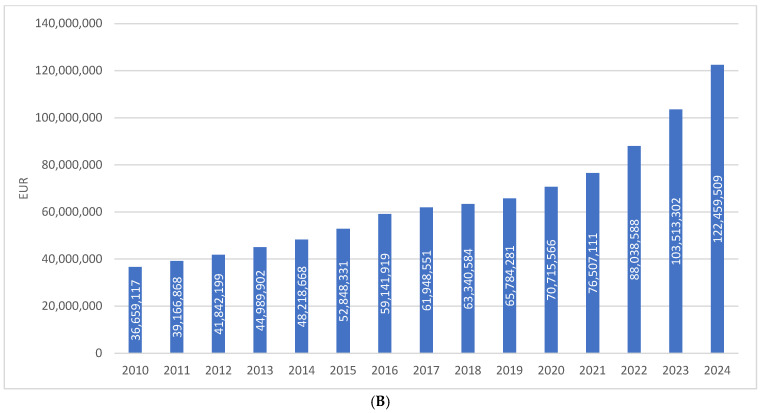
(**A**) The total use of A10 drugs from 2010 to 2024. (**B**) Financial expenditure of A10 drugs from 2010 to 2024.

The total consumption of drugs used in diabetes (ATC A10) in Croatia increased from 61.26 DDD/1000/day in 2010 to 117.97 DDD/1000/day in 2024, representing a growth of approximately 92%, or slightly less than twofold, over a 15-year period. In the same period, financial expenditure on A10 medicines rose from €36.66 million in 2010 to €112.46 million in 2024, which corresponds to an increase of more than threefold. This disproportionate growth in expenditure compared to utilization is attributable to the introduction of higher-priced antidiabetic agents into routine clinical practice (Figure 1A,B).

**Figure 2:**
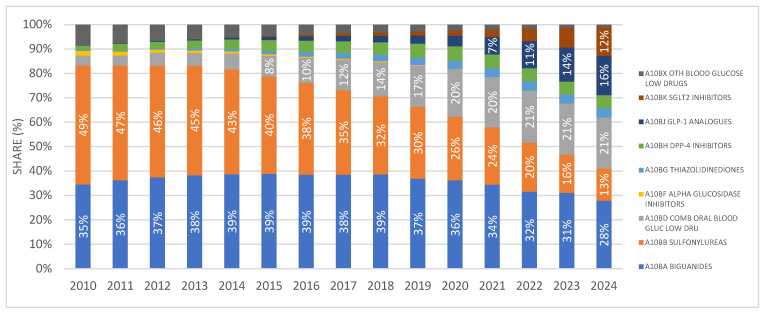
Shares of different antidiabetic drug classes in prescribing as DDD/1000/day. Note: the first SGLT2 inhibitor was introduced to the Croatian market in 2014.

The share of SGLT2 inhibitors as monotherapy in the prescribing of antidiabetic medications (excluding insulins) has been continuously increasing since their market introduction, reaching almost 12% in 2024. This places them after biguanides (28%), fixed-dose combinations (21%), GLP-1 receptor agonists (16%), and sulfonylurea derivatives (13%). The distribution is illustrated in Figure 2.

SGLT2 inhibitors were introduced to the Croatian market in 2014, and since then their prescribing has shown a continuous upward trend, increasing from 0.49 DDD/1000/day in 2014 to 11.63 DDD/1000/day in 2024. During the same period, the financial expenditure on drugs from this group ranged from €34,320 in 2014 to €21,878,396 in 2024.

**Figure 3:**
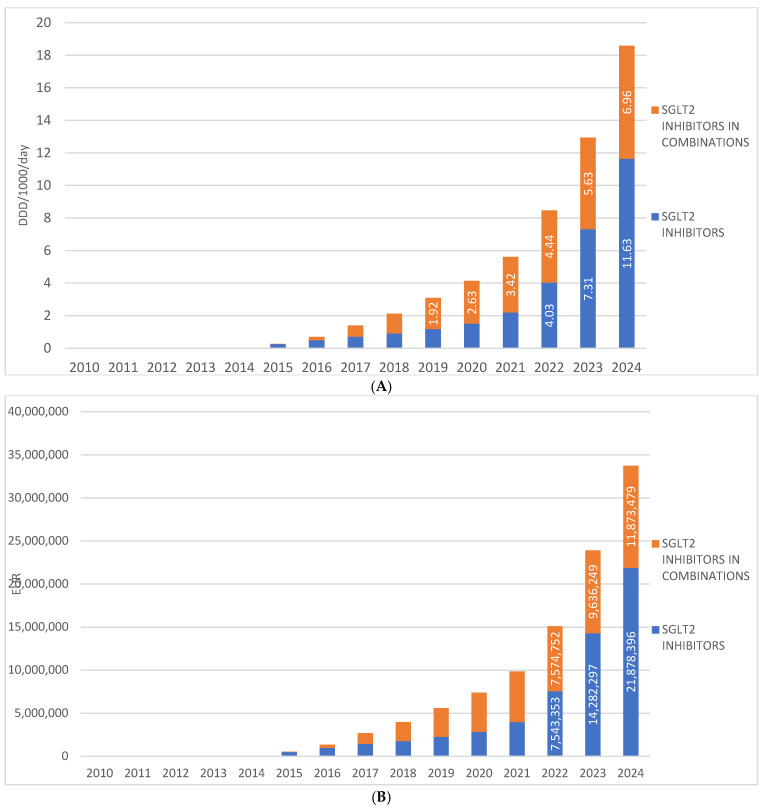
(**A**) The total use of SGLT2 inhibitors (mono and combinations) from 2010 to 2024. (**B**) Financial expenditure of SGL2 inhibitors (mono and combinations) from 2010 to 2024. Note: the first SGLT2 inhibitor was introduced to the Croatian market in 2014.

Fixed-dose combinations of SGLT2 inhibitors with other antidiabetic agents were introduced to the Croatian market one year after the introduction of SGLT2 inhibitor monotherapies, i.e., in 2015. In 2024, these combinations were prescribed at a rate of 6.96 DDD/1000/day, which is approximately half the utilization rate of monotherapy preparations. In 2024, the financial expenditure for fixed-dose combinations of SGLT2 inhibitors amounted to €11,873,479. (Figure 3A,B).

Among individual drugs within this group in Croatia, dapagliflozin is the most prescribed, having been introduced in 2014, with a utilization rate of 7.17 DDD/1000/day in 2024. The second most prescribed is empagliflozin, introduced in 2016, with a utilization rate of 4.46 DDD/1000/day in 2024. Ertugliflozin was present on the market from 2019 to 2022, with utilization ranging from 0.04 to 0.1 DDD/1000/day. Canagliflozin was available only in 2014 and 2015, with a utilization rate below 0.1 DDD/1000/day.

**Figure 4:**
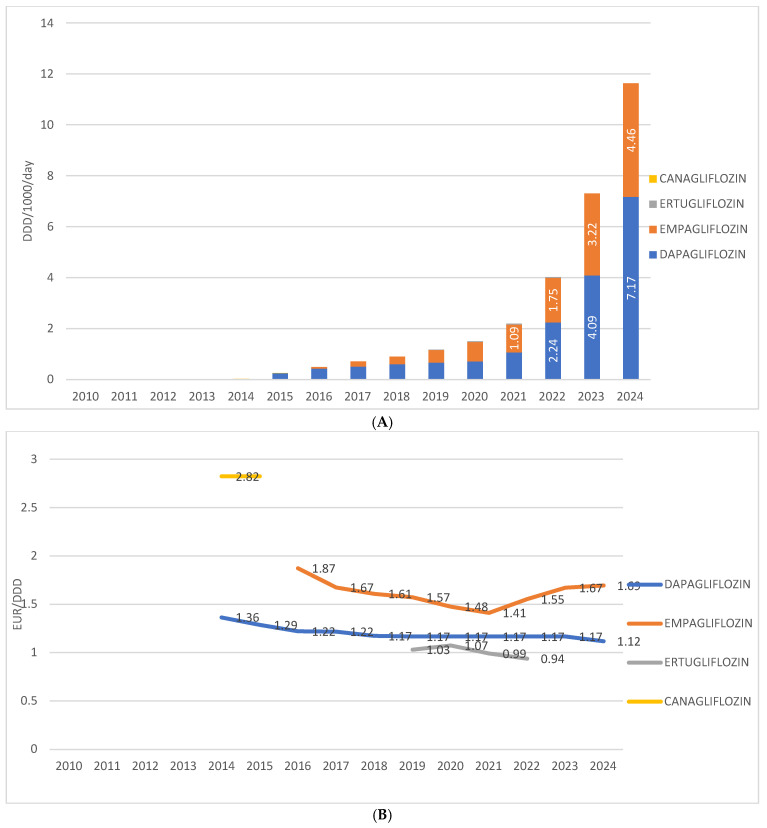
(**A**) Prescription of different SGLT2 inhibitors from 2010 to 2024. (**B**) Price per 1 DDD of different SGLT2 inhibitors from 2010 to 2024. Note: the first SGLT2 inhibitor was introduced to the Croatian market in 2014.

The price of the most prescribed SGLT2 inhibitor in monotherapy—dapagliflozin—ranged from EUR 1.36 per DDD in 2014 to EUR 1.17 per DDD in 2024. The price of the second most prescribed agent in this group, empagliflozin, decreased from EUR 1.87 per DDD at its introduction in 2016 to EUR 1.41 per DDD in 2021, followed by an increase to EUR 1.69 per DDD in 2024. The price of ertugliflozin varied between EUR 0.94 and EUR 1.07 per DDD, while in 2014, canagliflozin was recorded at a price of EUR 2.82 per DDD (Figure 4A,B).

**Figure 5:**
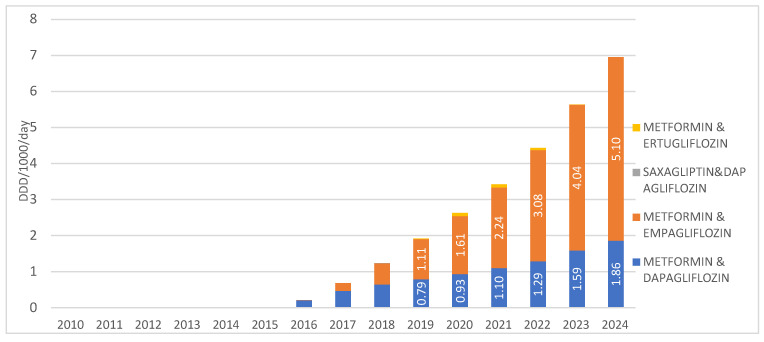
Prescription of SGLT2inhibitors in combinations from 2010 to 2024. Note: the first SGLT2 inhibitor was introduced to the Croatian market in 2014.

When observing SGLT2 inhibitors in combination with other medications, the highest prescribing rate is recorded for the combination of metformin with empagliflozin, introduced to the market in 2016, with a utilization rate of 5.1 DDD/1000/day in 2024. The second most prescribed combination in this group is metformin with dapagliflozin, introduced in 2015, with a utilization rate of 1.86 DDD/1000/day in 2024. The combinations of saxagliptin with dapagliflozin, as well as metformin with ertugliflozin, are registered on the Croatian market but show negligible utilization, less than 0.01 DDD/1000/day, and were not prescribed at all in 2024 (Figure 5).

The prices of these fixed-dose combinations of SGLT2 inhibitors with other medications remained generally stable during the observed period. The price ranged from €1.31 (in 2015) to €1.19 per DDD (in 2025) for metformin with dapagliflozin, while for metformin with empagliflozin, it was consistently €1.23 per DDD in all years since its market introduction in 2016 up to the present.

## 4. Discussion

This nationwide pharmacoepidemiological analysis reveals the accelerating uptake of SGLT2 inhibitors in Croatia over the past decade, with two distinct inflection points aligning with pivotal shifts in clinical guidance and evidence generation. One should bear in mind that, potentially on top of the guideline updates and emerging evidence we highlighted, factors such as national reimbursement changes, patent expirations, marketing authorization expansions, and COVID-19-related prescribing disruptions may also have influenced the observed inflection points.

Most existing European studies on SGLT2 inhibitors have focused on national prescription or sales data, which, while useful, do not necessarily reflect actual medication use since prescriptions do not guarantee drug dispensing or patient adherence. In contrast, our study is based on precise utilization data sourced from IQVIA database, capturing actual drug volumes distributed to pharmacies, thus providing a more accurate and real-world picture of SGLT2 inhibitor use. Moreover, unlike most prior research limited to diabetes indications and shorter timeframes, we included comprehensive data spanning all years since market introduction and encompassing all relevant indications, including heart failure and chronic kidney disease. For context, an observational study comparing antidiabetic drug consumption across five major European countries (Germany, Spain, France, Italy, and the UK) used retail sales data converted to DDD per 1000 inhabitants per day (DDD/TID), revealing distinct utilization patterns of SGLT2 inhibitors [21]. Notably, in France, SGLT2 inhibitor sales started only in 2020 but surged from an average of 0.12 DDD/TID per month (95% CI: 0.04; 0.20) in 2020 to 3.87 (95% CI: 3.12; 4.61) in 2022, a staggering 3125% increase within three years [22]. Similarly, in Hungary, national antidiabetic medication use rose to 94.8 DDD/TID by 2021, with SGLT2 inhibitor use dramatically increasing from 0.4 DDD/TID in 2015 to 6.8 DDD/TID in 2021 (*p* < 0.001) [23]. To the best of our knowledge, the present study breaks new ground by examining comprehensive utilization trends across multiple indications, making a unique contribution to European pharmacoepidemiological literature.

The first major increase in prescribing occurred in 2021, temporally aligned with the release of the 2021 European Society of Cardiology (ESC) guidelines for the diagnosis and treatment of heart failure [15]. These guidelines formally recommended SGLT2 inhibitors as foundational therapy for heart failure with reduced ejection fraction (HFrEF), based on robust evidence from trials such as DAPA-HF and EMPEROR-Reduced [8,9]. This was also the period when preliminary signals of nephroprotective benefit began to emerge from cardiovascular outcome trials (CVOTs), including EMPA-REG OUTCOME and DECLARE-TIMI 58 [7,24], which reported reductions in albuminuria and slowing of estimated glomerular filtration rate (eGFR) decline. These early renal findings—though secondary or exploratory—contributed to growing confidence in the broader organ-protective potential of this drug class.

The second pronounced surge was observed in 2023, coinciding with the focused update of the ESC heart failure guidelines [16]. This revision was driven by evidence from the EMPEROR-Preserved and DELIVER trials, which demonstrated significant benefits in patients with heart failure with preserved ejection fraction (HFpEF), a population historically underserved by pharmacotherapy [10,11]. Simultaneously, definitive nephroprotective evidence emerged from DAPA-CKD and EMPA-KIDNEY, which enrolled patients with chronic kidney disease, both diabetic and non-diabetic [12,13]. These findings not only expanded the therapeutic indications of SGLT2 inhibitors but also perpetuated updates in nephrology and diabetology guidelines [14,17,18]. The expansion of reimbursement criteria and prescriber awareness in Croatia likely contributed to the notable escalation in use during this time.

Among individual agents, dapagliflozin was the most frequently prescribed. Its preferential use may reflect a combination of clinical versatility, supported by efficacy data across glycaemic control, HFrEF, HFpEF, and CKD, as well as the economic factors [8,9,10,11,12,13]. Although empagliflozin demonstrated superiority in reducing major adverse cardiovascular events (MACE) in EMPA-REG OUTCOME [7], dapagliflozin’s consistent benefits across diverse endpoints and its lower average unit cost (currently 1.12 €/DDD vs. 1.69 €/DDD for empagliflozin) likely influenced prescriber behavior, particularly within budget-conscious systems.

To the best of our knowledge, despite the encouraging trend, underutilization remains a concern. SGLT2 inhibitors (in monotherapy) accounted for only 12% of non-insulin antidiabetic prescribing in 2024. Although evident, claims about therapeutic inertia, specifically in patients with high cardiovascular risk as well as non-diabetic heart failure or chronic kidney disease patients, cannot be directly confirmed in the present study due to the absence of indication-level data, prescriber specialty, and patient–diagnosis linkage. Therefore, our observations should be interpreted as indirect evidence suggestive of potential inertia, highlighting the need for further research with more granular data to confirm these findings. In the current therapeutic landscape, failing to prescribe SGLT2 inhibitors to eligible patients represents a missed opportunity for disease modification and event prevention, especially in patients without diabetes.

A further positive development is the increasing use of fixed-dose combinations, particularly with metformin. Such regimens are associated with improved adherence and therapeutic persistence, which in turn can enhance long-term outcomes and health system efficiency. The trend toward fixed combinations reflects a maturation of prescribing practices that align with real-world adherence challenges.

These findings reinforce the need for continuous audit and feedback, clinician education, and evidence-based guideline implementation. As SGLT2 inhibitors transition from niche use in diabetology to cornerstone therapy in cardio-renal-metabolic care, systematic efforts are needed to close the gap between evidence and practice. Addressing therapeutic inertia in this context is no longer a matter of glycaemic optimization alone (in those patients living with diabetes), but of holistic, organ-protective chronic disease management.

### Strengths and Limitations

This study has several limitations that merit consideration. Although the analysis is centered on Croatia, the breadth and depth of available data support a degree of extrapolations, offering findings that may hold relevance for broader international context. Key determinants of prescribing behaviors, such as changes in pricing policies, evolving therapeutic guidelines, emerging clinical evidence, and pharmaceutical marketing, can be identified and evaluated for their potential applicability within national healthcare systems to anticipate or influence prescribing trends.

The primary data source was IQVIA database, which aggregates sales information from all wholesalers supplying both public and hospital pharmacies across Croatia. This dataset reflects the volume of medicines distributed to pharmacies rather than prescriptions written or medications dispensed to patients. Consequently, the analysis may over- or underestimate actual patient use, particularly as fluctuations in pharmacy stock levels are not captured and are assumed to be relatively constant over time. Moreover, the use of the DDD methodology (expressed as DDDs per 1000 inhabitants per day) carries inherent limitations. DDDs represent the assumed average maintenance dose for a drug’s main indication and do not accommodate individual dose adjustments. Additionally, the retrospective application of the 2024 DDD definitions across the entire study period may in some points affect absolute consumption values and limit comparability with other studies using historical DDD standards.

While this remains the most robust and comprehensive measure of drug utilization currently available in Croatia, the inability to link data to individual patients nor diagnoses limits the analysis. As such, we cannot definitively attribute the observed rise in SGLT2 inhibitor use to improved patient access. Nonetheless, in the Croatian context, where universal health coverage is provided through the National Health Insurance Fund and all reimbursable medications are accessible to insured individuals, access to essential medications is unlikely to represent a significant barrier. Finally, the purely descriptive and ecological nature of the study design, without formal time-series analyses or inferential statistics, limits the ability to confirm temporal “inflection points” or draw causal inferences from observed trends. Although we indirectly point out some associations with guideline updates, no formal analyses support causal claims, so temporal trends should be interpreted cautiously. Wholesale sales data may not fully reflect actual patient use due to factors such as stockpiling, hospital versus outpatient use, parallel exports, and reimbursement delays, which are not accounted for in the data.

## 5. Conclusions

This national-level analysis demonstrates a marked and accelerating increase in SGLT2 inhibitor use in Croatia from 2014 to 2024, with two distinct inflection points coinciding with the 2021 and 2023 ESC heart failure guideline updates and the publication of landmark CKD trials (DAPA-CKD and EMPA-KIDNEY). Dapagliflozin was the most prescribed agent, likely influenced by its earlier market entry, marketing efforts, and lower cost relative to alternatives, rather than clinical superiority. Despite clear, guideline-supported benefits, SGLT2 inhibitors appear to be underutilized, particularly among patients not having diabetes with heart failure and especially those with chronic kidney disease. However, given the aggregated nature of sales data and lack of indication-level or patient-specific information, these findings should be interpreted cautiously.

The increasing use of fixed-dose combinations suggests a positive shift toward adherence-friendly regimens. Overall, these results highlight the need for continued physician education, interdisciplinary coordination, and further research to better understand and address potential gaps in the implementation of evidence-based therapies across the cardio-renal-metabolic continuum.

## Data Availability

Data Source—IQVIA. Analysis based on IQVIA Data. All rights reserved. Authors can be contacted upon reasonable request.
